# Fatal Gastrointestinal Hemorrhage in a Young Boy with Newly Diagnosed Metastatic Medulloblastoma on High Dose Dexamethasone

**DOI:** 10.1155/2014/478326

**Published:** 2014-11-13

**Authors:** Victor Wong, Nathalie Lefloch, John R. Crawford

**Affiliations:** ^1^Division of Hematology Oncology, Department of Pediatrics, University of California-San Diego, 3020 Children's Way MC5009, San Diego, CA 92123, USA; ^2^Department of Neurosciences, University of California-San Diego, USA

## Abstract

A 10-year-old boy with newly diagnosed metastatic medulloblastoma was placed on high dose dexamethasone and ranitidine prior to surgery. The child underwent subtotal resection and was discharged 5 days postoperatively with an uneventful hospital course on a tapering dose of dexamethasone and ranitidine. Over the next 2 days the patient complained of mild abdominal distension with flatulence, without pain, vomiting, or dysmotility. On follow-up in clinic 5 days after discharge, he had normal vital signs when he suddenly became pale and had loss of consciousness. Emergent computerized tomography of the head showed no acute hemorrhage and complete blood count revealed hemoglobin of 4.2 gm/dL. In spite of maximum resuscitation with copious blood products the patient died. Autopsy revealed evidence of duodenal perforation with intraluminal hemorrhage. This case demonstrates a rare fatal complication of high dose dexamethasone therapy even with concurrent gastrointestinal prophylactic therapy. We provide a review of the limited literature on steroid use in pediatric neurooncology with regard to gastrointestinal bleeding.

## 1. Introduction

Dexamethasone is frequently used in pediatric neurooncology to treat the vasogenic edema associated with tumor progression. The proposed mechanism is that the antiedema effect is obtained by reducing the permeability of tumor capillaries, through steroid effects on the cytoskeleton and cell-adhesion molecules, thereby preventing breakdown of the blood-brain-barrier [1]. There has been clinical benefit from steroids when used to treat patients with symptoms of increased intracranial pressure and edema, so much so that it has become the standard of practice to start high dose steroids perioperatively in patients with central nervous system (CNS) tumors. Though it is commonly used in patients with brain tumors when they are initially diagnosed and surrounding surgery, it can also be used with patients suffering from postoperative aseptic meningitis or to alleviate headaches associated with cerebral edema caused by radiation treatment. Dexamethasone has become the standard glucocorticoid of choice and carries the highest glucocorticoid potency. We present a rare fatal case of dexamethasone toxicity in a young child with newly diagnosed metastatic medulloblastoma to highlight the potential serious effects of this commonly used therapy in neurooncology.

## 2. Case Presentation

A 10-year-old boy with newly diagnosed metastatic medulloblastoma status after subtotal resection presented to follow-up neurooncology clinic for routine evaluation after recently being discharged from the hospital 5 days after surgery. The surgery itself was without complications despite being a subtotal resection. He was placed on perioperative dexamethasone (4 mg IV Q6 hours, equaling 0.458 mg/kg/day). The maximum dose of dexamethasone was chosen given the extent of his disease and concern for tumor associated edema, given that he had a subtotal resection. Ranitidine was added for gastrointestinal (GI) prophylaxis. Given that his course was uneventful, tapering began around discharge, and his dexamethasone dose was adjusted to 3 mg PO TID at discharge.

At the follow-up appointment, postoperative day 10 from surgery or 5 days after discharge, he reported transient abdominal distension with flatulence for the last 2 days, without any report of pain, nausea, vomiting, dysmotility, constipation, diarrhea, hematochezia, or melena. Review of past medical history and family history was noncontributory. The patient was on no other additional medications other than the dexamethasone and ranitidine. On physical exam, he was noted to be tired appearing with normal vital signs without any signs of abdominal distension and/or tenderness. Over the course of the visit he suddenly became pale and lost consciousness. Emergent head CT revealed no evidence of intracranial hemorrhage; however complete blood count revealed hemoglobin of 4.2 gm/dL. Despite rapid delivery of blood products the patient died in the intensive care unit less than 2 hours after presentation. An autopsy performed postmortem demonstrated extensive intraluminal blood within the intestinal tract without evidence of peritoneal hemorrhage or metastatic disease. There were extensive ulcerations in the duodenum on gross and microscopic analysis confirming the cause of death to be due to his gastrointestinal ulcer secondary to dexamethasone therapy.

## 3. Discussion

The dosing of dexamethasone at 16 mg/day in adults was actually established in the 1960s and there is no clear consensus on the optimal dose, duration, or tapering period in either children or adults in the neurooncology population [2, 3]. The therapeutic effects of dexamethasone have a subacute onset and marked neurological improvement can be seen within 24–72 hours after steroid initiation. In general once a patient shows clinical improvement dexamethasone should be weaned and discontinued, often in a nonuniform fashion. There are no evidence-based protocols for the weaning of dexamethasone and this is often left to the discretion of the treatment team.

With regard to the use of steroids in the management of CNS tumors, there have been very few controlled and documented studies in review of the literature. In the studies that have been published, significant improvement has been reported in symptomatic patients who receive steroids and when examining variable dosing between 4 and 16 mg/day, they showed no advantage with the higher dosing that is typically used. Published recommendations have stated that it is good practice to taper steroids within 1 week of starting therapy with the goal to discontinue within 2 weeks if possible. However, this was stated as Level 3 evidence-based recommendation, meaning that this was based on the report of the expert committee [4]. Review of Children's Oncology Group protocols do not list specific advice/guidelines with regard to the tapering of steroids and suggest that the dosing for dexamethasone should be 0.25–1 mg/kg/day divided every 4–6 hours (max of 16 mg/day) without mentioning of tapering schedule. It is likely that lower doses of steroids can be used in children than in adults. However to truly know the optimal dose, duration, and tapering schedule, these questions must be addressed specifically in future clinical trials.

There are many potential side effects associated with dexamethasone use. The specific side effects from dexamethasone use can be broken down into toxicity and/or withdrawal. Generally, the side effects related to toxicity include GI irritation, ulcers and bleeds, mood swings, weight gain, acne, impaired wound healing, and disrupted sleep. For side effects related to steroid withdrawal, this can be secondary to tumor with recurrent tumor associated edema. Additional side effects related to steroid withdrawal include symptoms of corticosteroid withdrawal syndrome that include nonspecific symptoms such as anorexia, weakness, nausea, vomiting, and anorexia. More serious and potential life-threatening side effect of withdrawal would be adrenal insufficiency [4]. Typically the risk of side effects from glucocorticoid use is dose-dependent, increases with duration, and is reversible with the discontinuation of steroids [2]. However, several reports have shown that risk of steroid toxicity can occur early in treatment, with a reasonable percentage within the first three weeks of initiation [5, 6]. The side effects of glucocorticoid use in neurooncology patients postoperatively have been documented, with similar complications that have been reported in patients who receive high dose dexamethasone for spinal cord compression as well [7].

Given that steroids are widely used and GI bleeds are a well-recognized complication, the diagnosis of bowel perforation in patients on high dose steroids can still be difficult and delayed. In the presentation of peritonitis, abdominal pain could be the only presenting sign. This is due to a presumed “masking effect” from glucocorticoids and appears to be dose dependent [5, 7]. Studies of various magnitudes have attempted to estimate the risk of GI bleed due to steroid use. One study examined the incidence of GI bleeding between different causative agents such as nonsteroidal anti-inflammatory drugs (NSAIDs), aspirin, and corticosteroids. They found that corticosteroids were associated with increased mortality; however there were confounding factors like comorbid conditions and inability to estimate the odds ratio given small sample size [8]. In one large meta-analysis, the authors concluded that there was an increased relative risk for developing GI bleeds in patients on corticosteroids and especially for patients on more than one medication [9]. However, like most studies, they did not specifically examine the risk of only within the neurooncology population and more specifically in the pediatric population. In a smaller case series that examined the incidence of GI perforation versus bleeding, specifically in a patient population that received dexamethasone for spinal cord compression, Fadul et al. reported that incidence of perforation versus bleeding was 2.8 versus 1.9%, respectively [10]. This case highlights an important taxonomy of GI complications, not previously mentioned, and that is the risk of bleeding versus perforation. Regardless of bleeding or perforation, the severity and seriousness of GI-related complications in patients treated with corticosteroids have been well documented in pediatrics, especially in preterm infants who receive steroids for the prevention of chronic lung disease and children affected with bacterial meningitis [11–13]. However, no reports were found in the pediatric neurooncology literature.

Given the potential for significant gastrointestinal complications associated with steroid use, it has become the standard of practice to prophylactically use H2-blockers such as ranitidine with high dose steroid administration. This practice has been adapted from our trauma/intensive care colleagues [14]. In the intensive care setting, other risk factors associated with stress-related upper GI bleeding in addition to corticosteroids include organ failure, trauma, acute central nervous system injury, NSAIDs, and sepsis [15]. The utility/effectiveness of GI prophylaxis while patients are on steroids has not been the focal point of any well-designed clinical trial. However, our case demonstrates that even with prophylactic gastrointestinal protection serious adverse effects can still occur.

Novel therapies are now being examined in the use of CNS tumor patients with hope of having steroid sparing effects. These include cyclooxygenase-2 inhibitors as well as VEGF antibodies, like bevacizumab [1]. There is even a recent report of a synthetic peptide formulation of corticotropin releasing factor that was developed as an alternative to dexamethasone that had entered in phase III study as a steroid sparing agent in adults with malignant brain tumors. Recht et al. reported a reduction in dexamethasone dose and improvement of corticosteroid-associated myopathy and that the patients who received the study drug were less likely to develop Cushing's disease [16]. There was no mention of GI complications. However, even with these novel agents, steroids remain the standard of care perioperatively. In one case report, Wheeler et al. reported complications with dehiscence of abdominal striae when the patient was treated with the combination of steroids and bevacizumab [17].

The side effects of steroids, specifically the risk of gastrointestinal hemorrhage, are common and can be a severe complication. From our literature review there are reports of pediatric gastrointestinal bleeding on steroids; however there are none in the neurooncology population. However, given this concern for GI bleeds, many if not most institutions have adopted the practice of using gastroprotective agents like H2-blockers and proton pump inhibitors as prophylaxis. Our case demonstrates that fatal gastrointestinal hemorrhage can occur even when every effort has been made to taper off high dose dexamethasone and even when the patient is on GI prophylaxis. More importantly from a clinical teaching point, the diagnosis of duodenal perforation can be difficult to diagnose in patients on dexamethasone and may occur in the absence of hematochezia and hematemesis in the early stages, as was the case with our patient.
